# Will an Unsupervised Self-Testing Strategy for HIV Work in Health Care Workers of South Africa? A Cross Sectional Pilot Feasibility Study

**DOI:** 10.1371/journal.pone.0079772

**Published:** 2013-11-27

**Authors:** Nitika Pant Pai, Tarannum Behlim, Lameze Abrahams, Caroline Vadnais, Sushmita Shivkumar, Sabrina Pillay, Anke Binder, Roni Deli-Houssein, Nora Engel, Lawrence Joseph, Keertan Dheda

**Affiliations:** 1 Department of Medicine, McGill University, Montreal, Canada; 2 Division of Clinical Epidemiology, Department of Medicine, McGill University and Health Centre, Montreal, Canada; 3 Lung Infection and Immunity Unit, Division of Pulmonology and UCT Lung Institute, Department of Medicine and Institute of Infectious Diseases and Molecular Medicine, University of Cape Town, Cape Town, South Africa; 4 Global Health, Department of Health, Ethics and Society at Maastricht University, Maastricht, The Netherlands; 5 Department of Epidemiology, Biostatistics & Occupational Health, McGill University, Montreal, Canada; Hopital Bichat Claude Bernard, France

## Abstract

**Background:**

In South Africa, stigma, discrimination, social visibility and fear of loss of confidentiality impede health facility-based HIV testing. With 50% of adults having ever tested for HIV in their lifetime, private, alternative testing options are urgently needed. Non-invasive, oral self-tests offer a potential for a confidential, unsupervised HIV self-testing option, but global data are limited.

**Methods:**

A pilot cross-sectional study was conducted from January to June 2012 in health care workers based at the University of Cape Town, South Africa. An innovative, unsupervised, self-testing strategy was evaluated for feasibility; defined as completion of self-testing process (i.e., self test conduct, interpretation and linkage). An oral point-of-care HIV test, an Internet and paper-based self-test HIV applications, and mobile phones were synergized to create an unsupervised strategy. Self-tests were additionally confirmed with rapid tests on site and laboratory tests. Of 270 health care workers (18 years and above, of unknown HIV status approached), 251 consented for participation.

**Findings:**

Overall, about 91% participants rated a positive experience with the strategy. Of 251 participants, 126 evaluated the Internet and 125 the paper-based application successfully; completion rate of 99.2%. All sero-positives were linked to treatment (completion rate:100% (95% CI, 66.0–100). About half of sero-negatives were offered counselling on mobile phones; completion rate: 44.6% (95% CI, 38.0–51.0). A majority of participants (78.1%) were females, aged 18–24 years (61.4%). Nine participants were found sero-positive after confirmatory tests (prevalence 3.6% 95% CI, 1.8–6.9). Six of nine positive self-tests were accurately interpreted; sensitivity: 66.7% (95% CI, 30.9–91.0); specificity:100% (95% CI, 98.1–100).

**Interpretation:**

Our unsupervised self-testing strategy was feasible to operationalize in health care workers in South Africa. Linkages were successfully operationalized with mobile phones in all sero-positives and about half of the sero-negatives sought post-test counselling. Controlled trials and implementation research studies are needed before a scale-up is considered.

## Introduction

A vast proportion of HIV infected individuals worldwide (6/10) do not know their serostatus [1]. This is because HIV testing in health care facilities are impeded by long wait times, lack of privacy and fear of loss of confidentiality of test results, and stigma and discrimination associated with an HIV diagnosis [2]–[4]. A self-testing strategy for HIV offers a private and confidential alternative to facility-based tests, but evidence on feasibility of self-testing from South Africa remains limited.

Knowledge of HIV sero-status with self-tests could influence risk-taking behaviours and impact risk reduction [5]–[7]. If self-tests could successfully be offered to proactive self testers with expedited counselling and referral to linkages, then, the strategy could potentially increase awareness of sero-status, and improve engagement of patients with health systems. Further, with timely treatment initiation, control of HIV infection and expanded access to HIV testing is a possibility [8]. However, observational research evidence is needed before trials are attempted.

South Africa has the world's highest global HIV/AIDS burden [9]. With an absolute burden of infection at 5.26 million of a total population of 10 million and an annual incidence in 15–49 age group of 0.85 m, it tops the world in HIV/AIDS statistic [10]. Despite a high endemicity of HIV infection, anecdotally only 50% of South Africans self report for having tested for HIV at least once in their lifetime [11]. This is largely due to the fact that the affected marginalized populations (i.e., migrant workers, women, commercial sex workers, men who have sex with men) who are at risk for HIV face social and structural barriers that hamper access to facility-based HIV testing. These barriers include fear of social visibility, fear of lack of confidentiality regarding test results, a lack of privacy associated with HIV testing in health care facilities, and rampant stigma and discrimination of HIV infected populations in these settings.

Despite availability of generic antiretroviral treatment, an early engagement to care is not yet a reality in South Africa. There is an urgent need to arrange for private and confidential alternatives to facility-based testing. To address this public health need, we developed an innovative, synergistic, HIV self-testing strategy that can be performed with oral HIV tests, and in future, in the comforts of one's own home. We evaluated this strategy in a population of health care workers (HCW) from the University of Cape Town. HCW are at a high risk of getting infected with HIV because of the nature of their profession, are anecdotally known to self-test for HIV, but less likely to engage in testing due to fear of loss of confidentiality, privacy and social visibility associated with facility based tests [12]. In this report, we describe the results from a pilot evaluation of this innovative strategy .

Self-testing is a process that requires a certain level of self-motivation to purchase the test, perform the test properly, interpret the self-test result accurately, and proactively seek linkages to counselling, confirmatory testing or treatment (as the case may be) by contacting a counsellor or health center, either in person or on phone or internet [13] Realizing that self-test conduct and interpretation were key to obtaining an accurate result, and linkages to treatment( in self test positives) or counselling (in self test negatives) were essential to the success of the strategy, we developed innovations to support these aspects.

In a recent systematic review, we summarized global evidence on the two self-testing strategies that have been evaluated worldwide [13]: a) a supervised self-testing strategy, and b) an unsupervised self-testing strategy [13]. In a supervised self-testing strategy, participants perform the self-testing process by themselves in a private kiosk set up in a clinic or outreach facility, with assistance offered at any time by a counsellor or health care professional, that are available on site. In contrast, in an unsupervised self-testing strategy, the self-testers not only perform, interpret and record the HIV self-test themselves, but also proactively seek linkages to counselling and treatment (over the phone, internet or face-to-face) facilitated by counsellors potentially available 24/7/ [14]. While global evidence on the supervised self-testing strategy exists, evidence on the unsupervised self-testing strategy is non-existent especially from Southern Africa [13].

In this report, we present results from a pilot cross sectional study that evaluated an unsupervised self-testing strategy evaluated in HCWs. We primarily estimated feasibility of the self-testing strategy defined by the completion rate (i.e. completion of self-test conduct, linkages to ART in seropositives and risk reduction counselling in seronegatives). Our secondary objectives were to: 1) evaluate the self-test's accuracy, 2) document participant preferences, concerns and experience with the self-testing strategy, and, 3) evaluate HIV sero-positivity.

## Methods

Two Institutional Review Boards based at McGill University Health Centre Montreal, Canada and at the University of Cape Town, South Africa approved the study. Written informed consent was obtained from all participants. Research was conducted in accordance with the principles expressed in the Declaration of Helsinki.

**Figure 1:**
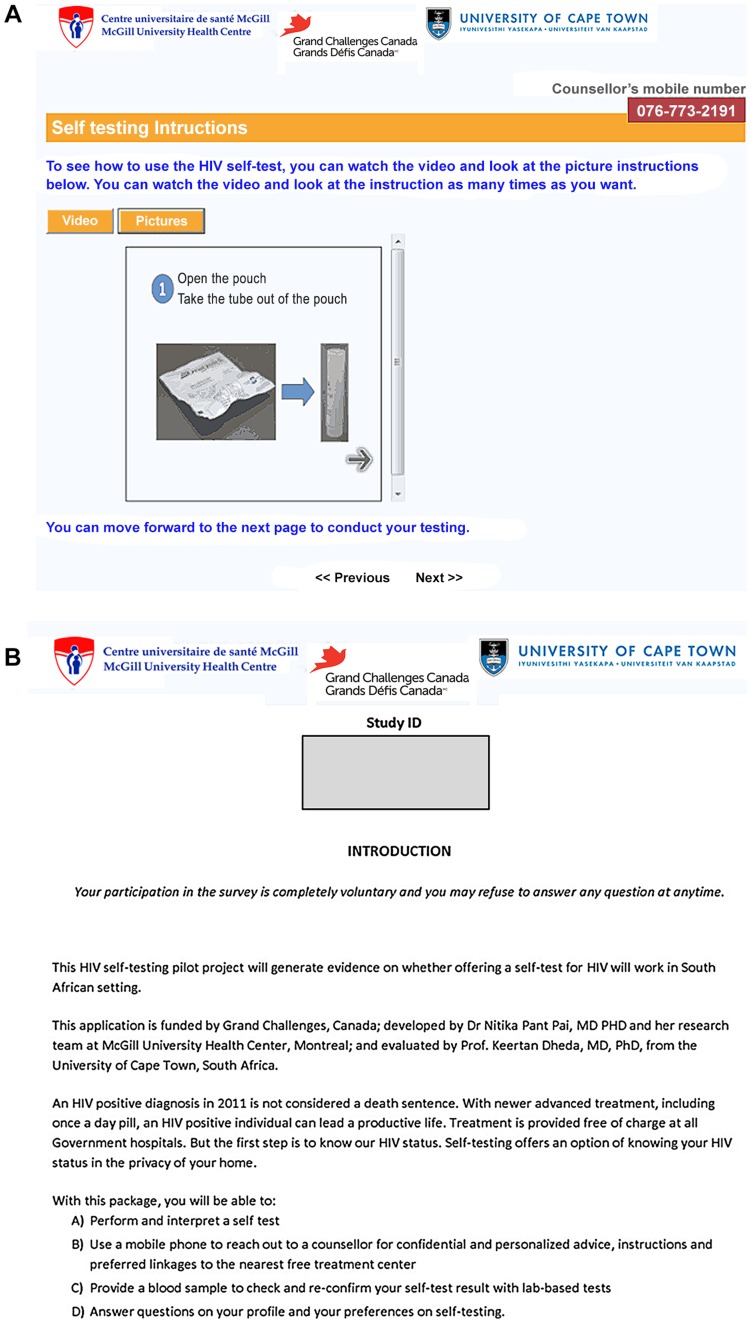
Applications. a) Internet- based b): Paper-based.

Our innovative strategy consisted of two self-testing applications; internet-based and paper-based (refer Figure 1). These were synergized with counsellors who offered post-test counselling and arranged for expedited referrals over phones. Our applications included: a) an oral HIV rapid point-of-care (POC) test approved for use in South Africa (OraQuick Rapid HIV-1/2 Antibody Test, OraSure Technologies, Inc., PA, USA); b) pre-test counselling and information on HIV; c) Self-staging questions for HIV risk that generated a self-rated HIV risk score of high, medium or low risk for HIV; d) instructions to self-test (i.e., videos, pictures); e) linkages to post-test counselling, over the phone or face-to-face (if preferred) operationalized confidentially ; f) anonymized self-test confidential record and interpretation on the application, and lastly g) anonymized data collection on demographics, risk profile, preferences and concerns with self-testing.

Between January and June 2012, we enrolled 251 HCW (e.g. doctors, nurses, lab technicians) in a cross-sectional study at Groote Schurr Hospital, a tertiary care centre in Cape Town, South Africa. HCWs who spoke English, were 18 years of age or older, of undocumented HIV sero-status at baseline (i.e. the participant was unaware of his/her sero-status) and were able to provide informed consent were recruited. This population was chosen because: a) HCWs are at risk for HIV occupationally and are anecdotally known to self-test, and, b) they may benefit from a private HIV self-testing option that averts perceived stigma and discrimination associated with seeking an HIV test in the work place, and lastly, c) the population was digitally literate, and desired to try out an unsupervised self-testing strategy. The study was funded by Grand Challenges Canada. The oral test used in our study, Oraquick (R) HIV1/2 (i.e., Orasure technologies, PA, USA) is an FDA approved, CE marked test that is approved for sale and use in South Africa.

Study flyers and pamphlets were pasted across the hospital campus with the phone number and interested participants contacted the research nurse. A convenient sample of HCW who showed up and consented to the study procedures was recruited. Participants were offered a choice of self-testing applications over the Internet and on paper. They chose the Internet or paper-based application according to their comfort and preference levels.

After informed consent was sought, the research nurse led participants to a private room equipped with a computer with Internet access, a landline and a mobile phone, and an oral self-test. Our internet-based self-testing application was administered online. (Refer figure 1) At any time throughout the testing process, an HIV counsellor was available to the self-tester. The self-testing application walked the participants through various stages of self-testing process similar to what they would experience in a health care facility, with the exception that the testing process was unsupervised and confidentially conducted with assistance sought over the phone or face-to-face if desired. The internet application consisted of HIV information (pre test counselling), a risk score section where one could stage one's own risk for HIV, a section that described the ideal conduct of a self-test, a section on interpretation of test lines, recording results of one's own self test result followed by a section on seeking linkages and responding to questions about the application. Linkages were sought by a confidential mobile phone call to a counsellor for post-test counselling and for referral and treatment linkages. Mobile phones and phone number were provided for this purpose. All the data was confidentially collected online and de-identified.

The paper based application (refer: Figure 1) and process was identical to the Internet application, except that instructions and information were pictorially represented and results were documented on paper.The participants who chose the paper application were also led by the research nurse to a private room to perform their self-tests. Linkages were confidentially sought by calling the phone number provided to them.

In parallel, blood was drawn on site from each participant for confirmatory rapid and laboratory based testing and sent to the reference laboratories linked by Study IDs for confirmatory testing. A standard National Health Laboratory Services (NHLS, South Africa) approved HIV testing algorithm was used for confirmatory testing (i.e., ELISA with p24 Antigen tests (Architect HIV Ag/Ab Combo, Abbott Laboratories, Wiesbaden, Germany) and Western Blot for positives). Additionally, study ID linked rapid HIV tests SD Bio line HIV Rapid Test [Anti-HIV 1/2] (Standard Diagnostics, Inc., Kyonggi-do, Korea) and Determine™ HIV 1/2 Ag/Ab Combo (Alere, Waltham, MA, USA) were performed on site.

Participants who tested positive with in-house rapid tests were flagged for expedited confirmatory laboratory testing and results were made available within 8 hours. All the participants were encouraged to call the counsellor for post-test counselling. Additionally, all house tested (rapid test) sero-positives, after receipt of expedited lab confirmed sero-positives were contacted by the counsellors to communicate and confirm their test results, offer post-test counselling, advice for ART staging and arranged for confidential referrals to a clinic of the participant's choice. All these services were operationalized using mobile phone calls and text services.

Lab confirmed test results for participants that tested negative with in-house rapid tests were available within the next working day. All self test negative participants who called were offered risk reduction counselling. Data was confidentially collected online using our Internet application and was exported into an EXCEL sheet linked by study ID. Data from paper-based application were manually entered in an EXCEL file. Both the data sets were merged and then analyzed in STATA version 10 (STATA Corp, Texas, USA). With a hypothesized completion rate of the self-testing process at 80%, a sample size of 251 gave us 95% confidence interval (CI) widths of +/−0.049. Data on demographics and risk factors were reported as proportions with 95% CI.

## Results

**Figure 2:**
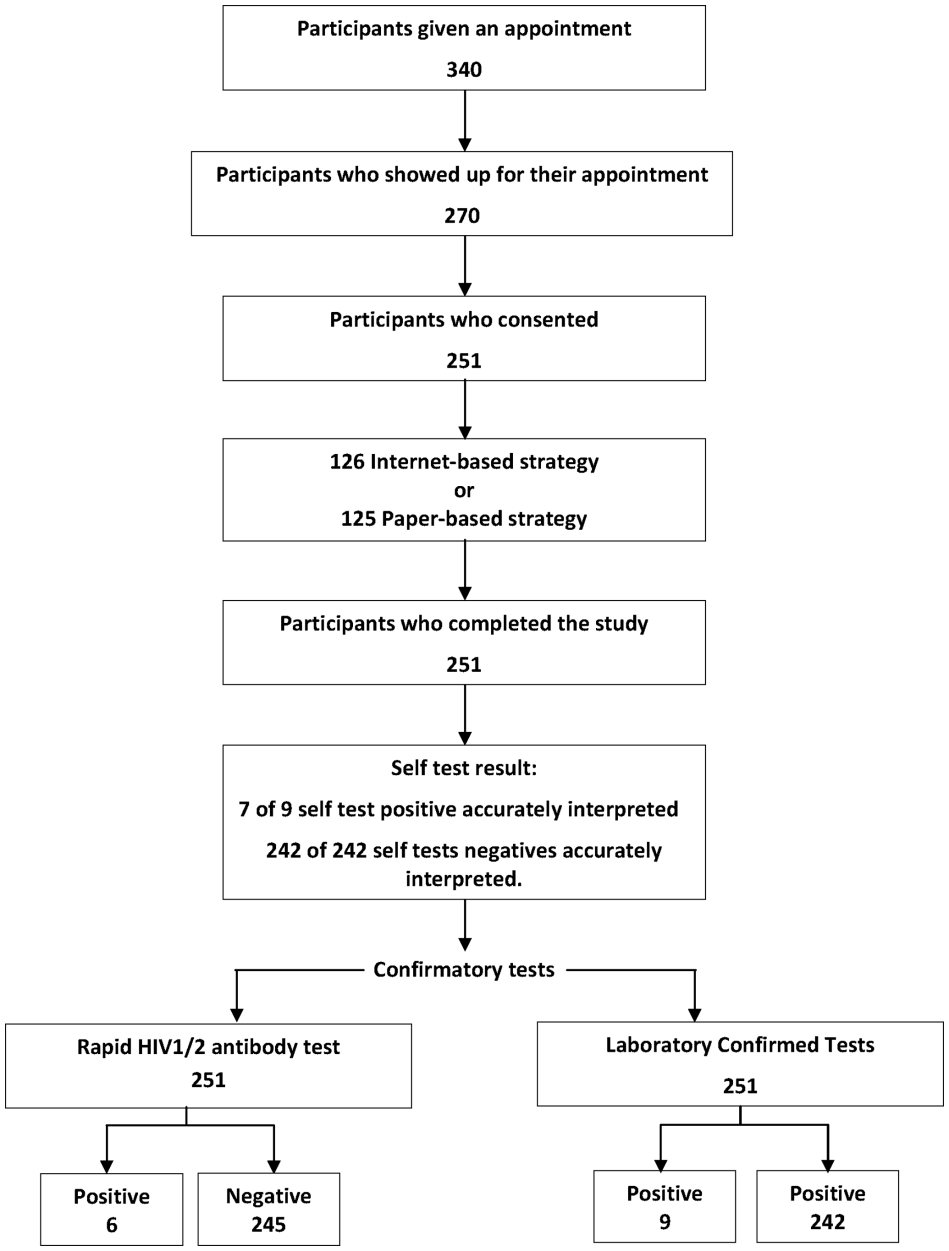
Flow of participants and testing algorithm.

Of 270 participants that were approached for study participation, 251 (93.0%) consented, of which 126 completed the internet-based application, and about 125 completed paper-based application (Figure 2). Of 251 study participants, 249 (99.2%) completed self-test interpretation and conduct successfully, giving us a completion rate of 99.2%.

About nine positives were identified, of which six accurately interpreted their self-test result. The other three participants' reported their self-test result as ‘negative’ whereas, the in-house rapid and confirmatory tests results were ‘positive’. The research nurse called the participants after the receipt of lab confirmed results as per IRB protocols. Their oral self-tests were performed again. On repeating these tests, two of the three participants self reported having seen a faint positive line on initial testing. On repeat testing, the faint positive lines persisted and were confirmed by the nurse to be positive. This suggests that self-testers may fail to read/interpret a faint or weak positive line as a positive self-test. At that time, our application did not have any information on the interpretation of faint positive lines. We incorporated the fact that any line (faint, strong or dark) was to be read as a positive line after completion of the study. The sole participant who was missed by a self-test was a false negative, who correctly interpreted his/her self-test result as negative with the antibody test, and the test failed him/her. This was because of a lack of p24 antigen in the test device that is antibody based that fails to detect sero-conversion. Information on this issue was included in the application.

In terms of linkages, through the use of mobile phones and confidential text messaging, all nine sero-positives received post-test counselling before ART staging and initiation, and were offered expedited and confidential linkage to care to a clinic of their choice. Therefore, our completion rates for linkages in nine seropositive were: 100% (95% CI, 66.0–100). Likewise, in 242 sero-negatives, about half of sero-negatives called for counselling on mobile phones, giving us a completion rate for linkages in sero-negatives at 44.6% (95% CI, 38.0–51.0).

HIV prevalence (sero-positivity) in our study sample was 3.6% [95% CI, 1.8–6.9]. With nine seropositives and 242 seronegatives, the sensitivity of reading self-tests compared to lab confirmed test results was 66.7% (95% CI, 30.9–91.0), and specificity was 100% (95% CI, 98.1–100), However, our positive predictive value (PPV) remained at 100% (95% CI, 51.7–100) and our negative predictive value (NPV) was at 98.8% (95% CI, 96.2–99.7). Sensitivity of self tests could certainly be improved with a call for development of antibody and antigen combined self tests that can quickly detect sero-conversion. One of these tests (Determine Alere Antibody/Antigen) combo test that is recently approved was used by us as in house rapid test.

Demographically, a majority of our participants, (78.1%; 196/251), were females. About 61.4% (154/251) were aged 18–24 years, and (54%; 135/251) had completed high school. For further details on demographics, refer to Table 1. Despite there being three facility-based HIV testing sites within the hospital campus, 63.1% (157/251) of participants had not sought HIV testing within the past six months and a further 33 (13%) participants had never ever tested for HIV in their lifetime. About 78.5% (197/251) of participants were sexually active and about 60% (150/251) self-reported having had unprotected sex within the past six months. About 8.8% (22/251) reported a history of needle-stick exposure and about 3.2% (8/251) self-reported a history of sexually transmitted infections. For further details on risk factors, refer to Table 2.

**Table 1:** Demographic profile of study participants.

Gender	Female	196 (78.1)
	Male	53 (21.1)
	Do not wish to answer	2 (0.8)
Age (years)	18–24	77 (30.7)
	25–34	77 (30.7)
	35–44	43 (17.1)
	>44	54 (21.5)
Education	Did not complete high school	15 (6.0)
	High school	135 (53.8)
	College or technical school	61 (24.3)
	Undergraduate degree	20 (8.0)
	Graduate degree	6 (2.4)
	Other	12 (4.8)
	Do not wish to answer	2 (0.8)
Occupation	Medical Professionals^1^	128 (51)
	Staff^2^	123 (49)

1 Attending physicians, residents, fellows, medical students, nurses, nursing students, paramedics.

2 Administrative staff, audiologists, cleaners, clerks, drivers, engineers, laboratory technicians, porters, security.

**Table 2:** Sexual and Behavioral Risk Factors of study participants.

Have you ever been tested for HIV?	Yes, in the past 6 months	93 (37.1)
	Yes, >6 months ago	124 (49.4)
	No, never been tested	33 (13.1)
	Do not wish to answer	1 (0.4)
If never been tested, why? (Multiple answers)	Not at risk of getting infected with HIV	13 (39.4)
	Don't want medical records to show my HIV status	5 (15.2)
	Don't want to know my HIV status	6 (18.2)
	Don't want to show up for HIV testing at a clinic	4 (12.1)
	Other	6 (18.2)
	Do not wish to answer	2 (6.1)
Are you sexually active	Yes	197 (78.5)
	No	26 (10.4)
	Do not wish to answer	28 (11.2)
Number of sexual partners in the past 6 months	0	34 (17.3)
	1	140 (71.1)
	2–5	17 (8.6)
	6–10	2 (1.0)
	>11	2 (1.0)
	Do not wish to answer	2 (1.0)
Have you had unprotected sex in the past 6 months?	Yes	150 (76.1)
	No	44 (22.3)
	Do not wish to answer	3 (1.5)
In the past 6 months, I have had sex with (multiple answers)	HIV infected partner	3 (1.5)
	Commercial sex worker	3 (1.5)
	Under the influence of alcohol	18 (9.1)
	Under the influence of drugs	3 (1.5)
	None of the above	165 (83.8)
	Do not wish to answer	7 (3.6)
In the past 6 months, have you injected drugs?	Yes	7 (2.8)
	No	243 (96.8)
	Do not wish to answer	1 (0.4)
Ever had a needle-stick exposure from a patient suspected to be HIV positive?	Yes	22 (8.8)
	No	228 (90.8)
	Do not wish to answer	1 (0.4)
If “yes”, after this exposure, did you get tested?	Yes	17 (77.3)
	No	4 (18.2)
	Do not wish to answer	1 (4.5)
Have you had a sexually transmitted infection?	Yes	8 (3.2)
	No	237 (94.4)
	Do not wish to answer	6 (2.4)
Perceived risk of getting infected with HIV	High	17 (6.8)
	Medium	52 (20.7)
	Low	163 (64.9)
	Do not wish to answer	19 (7.6)

Regarding self-testing experience, 91.2% (229/251) rated the experience as ‘very good’ or ‘good’ because of the privacy it offered, ease of use and non-invasive and painless nature of oral testing. For counselling, face-to-face counselling was an option acceptable to most participants (68.4%, 160/234), followed by technology enabled counselling (via mobile phone, Internet or text messages) at 40.6% (95/234) for post-test counselling, linkages and referrals.

On cost preferences, 93.2% (234/251) were willing to buy the oral self-test, with 57.7% (135/234) ready to pay R 1–50 (USD 0.10–6.30).

Other responses to our open-ended questions regarding concerns and challenges have been summarized in Table 3.

**Table 3:** Participant feedback to open ended questions.

Reflections on self-testing experience	
**Test Administration**	*“Test is easy and simple to use, results are easily interpreted”*
	*“I had a good experience having to test myself and that no blood was needed or needles involved during the test”.*
	*“I think this is a very good self-testing programme computer wasn't that bad but everybody don't have access or can use a computer.”*
**Privacy**	*I think it is good that they've come up with a test like this, now I can do it in the privacy and comfort at home.”*
**Factors influencing stress**	*“ I feel that this is a great way of testing because many people fear the whole process of being tested at the clinics”*
	*“It feels uncomfortable to check on my results. It is better if somebody told you.”*
	*“When I was doing the self-testing I was a bit nervous because I know that I had a high risk of being HIV positive.”*

Views on knowing status and personal risk	
**HIV status**	*“I feel confident knowing my HIV status”*
	*“It was a challenging experience but also gives you peace of mind in a way after the results”*
	*“It's a good device especially for people who are often very scared of doing their HIV test at clinics, but for other people who would take their HIV positive status very hard it would not be a better option, because that person can kill themselves at home if they are alone.”*
**Personal risk**	“*It opened my eyes because I thought I was very safe but now I know I am HIGH RISK and I don't think the questions are too personal. They are straight forward.”*
	*“Questions were relevant and straight to the point, they helped me to understand more about the risks associated with HIV”*

Views on taking self-testing public	
**Making HIV self-testing public**	*“It is a good idea and I think this method should also go to people that are in the communities and also in schools and it must be available in the pharmacy.”*
	*“Self-testing is great. But it would be better, if you sell it, to give a number to the person that will buy - the number of the counsellor in case of what the results will be and how will he/she handle them, before testing, or they can use a pharmacist to counsel the buyer”*
**Promotion of HIV self-testing**	*“I found the test interesting – will refer it to my friends and colleagues”*
	*“…I feel that more people will be willing to get tested now in the privacy of their own home as many people are concerned about going to a healthcare practitioner or facility”*

## Discussion

We evaluated an unsupervised self-testing strategy using an Internet application and its paper equivalent in a population of HCWs, who wanted to learn about their HIV status with an oral self-test and a novel approach. Despite their occupational risk for HIV, and despite their residing for years in a high endemic setting, a substantial proportion (63%) of participants reported not seeking an HIV test in the past six months. Our confidential self-testing strategy was well received, with 100% linkages for sero-positives operationalized within 24 hours and post-test counselling for sero-negatives offered to 44.6% participants. Additionally, a high proportion of participants (91%) reported an overall positive self-test experience. Similar findings on acceptability and preference have been reported in various other studies that have evaluated self-testing strategies [12], [14]–[18]. However, the completion rate for linkages of seropositive reported in our study is the highest reported for an un-supervised self-testing strategy. As an example, the completion rate for linkages is sero-positives in a US trial was about 96% [15].

Completion rate of linkages in sero-negatives was lower compared to sero-positives at 44.6%, but higher than the 29.2% statistic traditionally reported from other facility-based testing projects [19]. A high completion rate overall for both positives and negatives, found in our pilot study, could be ascribed to offering a confidential phone-based counselling strategy and use of an internet and paper-based applications that de-identified study participants. These results suggest that a majority of those participants who are found to be self-test sero-positives will possibly seek linkages to confirmatory testing, staging and care if they are expedited, privately arranged, and offered in a personalized manner with their preferred counselling option.

Results however need to be confirmed in other at risk populations such as men who have sex with men, commercial sex workers, and young pregnant women that have a high HIV burden that may also desire confidential and personalized self-testing. Linkage data for self-testing initiatives are currently limited; therefore the study adds new important data from South Africa.

Regarding diagnostic performance, the PPV, NPV, and specificity values of the self-test were high, but due to the low number of true self-test positives, our sensitivity estimates were low. Three self-test ‘negative’ participants who were contacted later voluntarily showed up for re-testing. Upon re-testing, two of three ‘negative self tests’ were discovered to have had faint positive lines self interpreted as ‘negative’ thus, highlighting the possibility of user errors in conduct and interpretation. In our study id linked positive sero-status was confirmed with the in-house rapid test and additionally, with laboratory confirmed test results, which were given by the research nurse to the participant. Interpreting faint positive lines was not included in our initial versions of self-testing applications, and this may have impacted participants' interpretation of a line. Interestingly, faint lines were also recently reported in the FDA document for approval of the self-test in the United States [15]. Following that, we improved our applications to state that any line with the self-test should be interpreted as a positive line. Although our sensitivity estimates improved with re-testing, to 88.9% (95% CI, 50.7–99.4), this phenomenon could occur in scale up initiatives.

Besides, expedited confirmatory and in house rapid testing also picked up the lone self-test negative tester that was not picked up by the oral antibody-based test because of sero-conversion. To further analyze this finding, we know that the oral antibody-based self-test is very sensitive at and after 90 days, therefore, the self-testers must be encouraged to re-test themselves after 90 days, particularly, if there is a high suspicion of a recent exposure, or a perceived risk of having an acute infection but the self test is found to be negative in these situations.

To conclude, the occurrence of user errors and inability of the test to pick up early/acute infection highlights the importance of providing clear instructions to the self-testers including limitations of self tests. Clear instructions on paper, the internet or on smartphone applications facilitate interpretation and enhance accurate performance of self-tests. Instructions will help avoid many errors in future. It is important to educate the public about the limitation of antibody-based test [20]. Therefore, to ensure the success of self-testing, messages regarding an accurate interpretation of positive lines and the limitation of the ability of the test to detect new infections within 90 days must at all times be emphasized.

**Figure 3:**
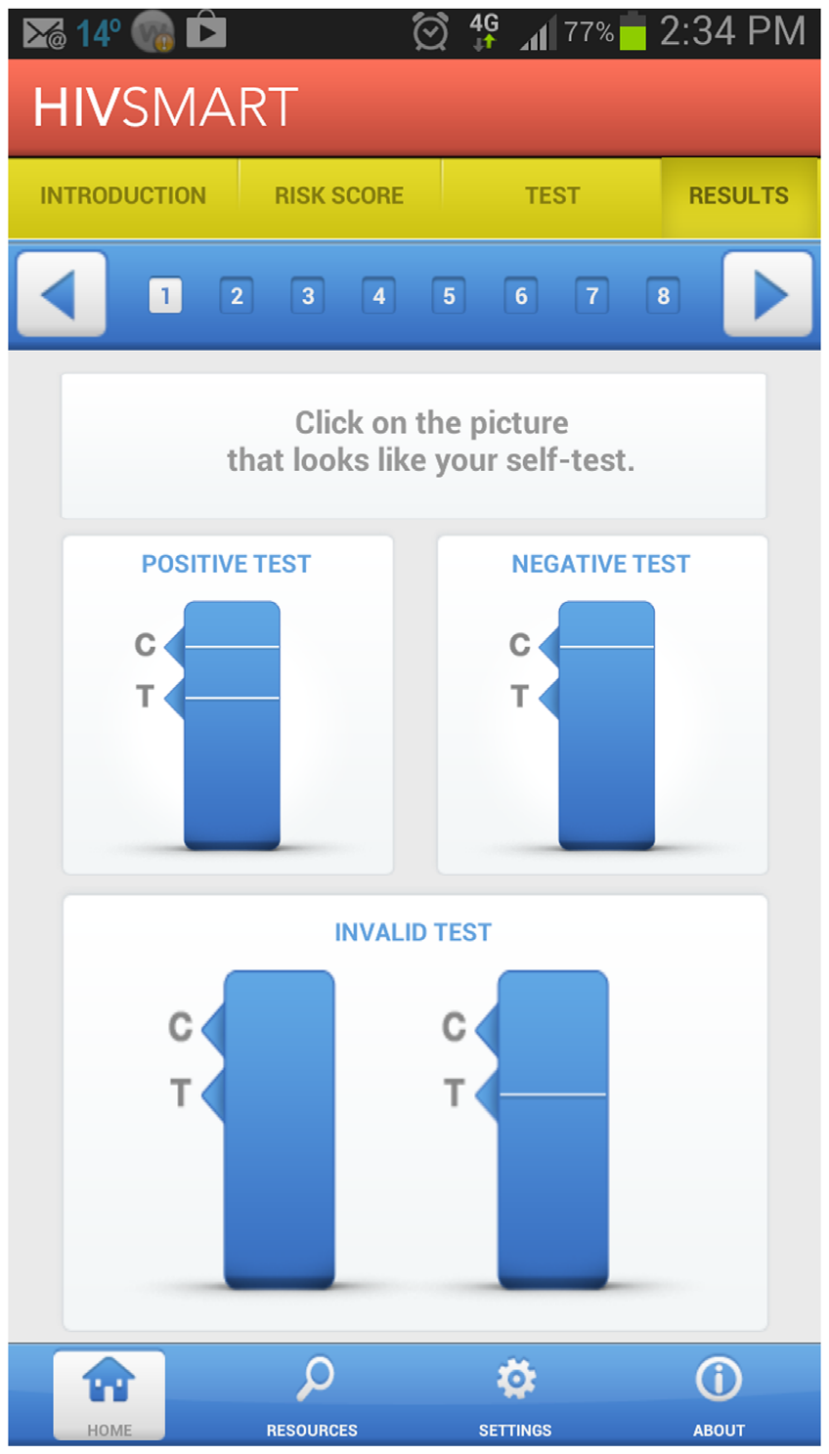
A HIV self-testing Smartphone Application (McGill University copyright no: 1105598).

At the FDA deliberations over approval of self-tests, arguments of access to HIV testing, won over arguments of sensitivity. Additionally, we have recently reviewed global evidence on the accuracy of these oral rapid antibody tests in an implementation research context. We found them to be 98.03% sensitive (95% CI, 95.85–99.08) and 99.74% (95% CI, 99.47–99.88) specific in research settings [20]. We converted our tested Internet application to an Android smartphone application (refer to Figure 3). This android application is available in English and could be downloaded to the Android phones. The smartphone application assists in understanding the process of self-testing and facilitates confidential linkages. This award winning application is copyrighted (no 4231105598), and owned by McGill University. Lastly, although successful use of internet-based strategies have been reported for sexually transmitted infections, and for HIV in global settings. But, to the best of our knowledge, no pilot study has synergized Internet, public health counsellors and mobile phones with an oral HIV test, and offered an integrated unsupervised self-testing strategy [21]–[25]. Our strategy offered complete confidentiality and anonymity in testing – a fact greatly appreciated by all the study participants.

### Limitations

This pilot study was conducted in a convenient sample of HCWs who consented to the evaluation of an unsupervised oral fluid based self-testing strategy. Selection and volunteer bias are likely. Misclassification of self-test results could lead to a potential for information bias.

Although the pilot study was successful, it needs to be repeated in other at risk populations to generate stronger evidence for feasibility of this strategy. These studies are being planned in scale up of this project. The study results are generalizable to HCWs who would show up for participation if a supervised self-testing strategy were offered again at any of the hospitals. Future scale up studies that evaluate innovative interventions in at risk populations such as men who have sex with men, pregnant women, commercial sex workers and adolescents will serve to generate stronger evidence of generalizability to at risk populations. Although in a high endemic setting, one could argue that routine HIV testing should be conducted in all populations and more so in populations that are at risk in hospitals and other health care settings.

## Conclusion

To conclude, self-testing offers a discreet, viable, private, option to test provided confidentiality is maintained and expedited linkages within a reasonable time window are made available. Our study demonstrates that linkages can be operationalized confidentially, and successfully with phones. Although our innovative, un-supervised self-testing strategy was well received by HCWs in South Africa, larger real life implementation research studies and controlled trials on self-testing are urgently needed to generate stronger evidence for scale up. Following up on a recent call from the WHO for pilot feasibility studies, this study has generated data on feasibility. Our findings have implications for future planning of scale up studies for Southern African region and similar regions and settings in Asia.
